# Association of different blood groups in patients with Dengue fever and their relationship with the severity of the illness

**DOI:** 10.12669/pjms.39.5.7275

**Published:** 2023

**Authors:** Naveed Iqbal, Muhammad Abdur Rahman Afridi, Zafar Ali, Adnan Rafiq

**Affiliations:** 1 Naveed Iqbal, FCPS. Department of Medicine, Lady Reading Hospital, Medical Teaching Institution, Peshawar, Khyber Pakhtunkhwa, Pakistan; 2 Muhammad Abdur Rahman Afridi, FCPS Medicine. Department of Medicine, Lady Reading Hospital, Medical Teaching Institution, Peshawar, Khyber Pakhtunkhwa, Pakistan; 3 Zafar Ali, FCPS. Department of Medicine, Lady Reading Hospital, Medical Teaching Institution, Peshawar, Khyber Pakhtunkhwa, Pakistan; 4 Adnan Rafiq, FCPS Trainee. Department of Medicine, Lady Reading Hospital, Medical Teaching Institution, Peshawar, Khyber Pakhtunkhwa, Pakistan

**Keywords:** Blood Groups, Dengue Virus, Dengue Fever, Dengue Hemorrhagic Fever, Dengue Shock Syndrome

## Abstract

**Objective:**

To determine the association of different blood groups in patients with Dengue fever and their relationship with the severity of the illness.

**Methods:**

A hospital-based descriptive study was conducted in the Dengue Isolation Ward of Lady Reading Hospital Peshawar from March 2020 to September 2020. Patients with Dengue fever were included in the study. The severity of the illness was categorized as “Dengue fever (DF)”, “Dengue hemorrhagic fever (DHF)”, and “Dengue shock syndrome (DSS)”. The patients’ blood groups were determined as A, B, AB, and O groups. All the data were recorded and analyzed using SPSS® version 23. Chi-square (*χ^2^*) and student *t*-test were applied, and a *p*-value of ≤0.05 was considered significant.

**Results:**

Out of 160 patients, 119(74.4%) were males; the patient’s mean age was 38.09±15.68 SD, IQR=25 years. Greater proportion (28%) of the young men (up to 40 years) was affected compared to 9% young women. Fever (99%) and body aches (96%) were the most common presentation of DF, complicated by bleeding in 30.6% and shock in 9.4% of the patients. The majority (63.1%) of the patients had DF; 27.5% had DHF, and 9.4% had DSS. Sixty three (39.4%) patients had blood Group-B and 5.6% had Group-AB (*p*=0.97). The proportion of patients with different blood groups and the type/severity of the DF were almost identical except the fact that none of the patient with group AB had DSS. There was significant gender difference of hemoglobin (*p*=0.008, 95%*CI*=0.439, 2.844), hematocrit (*p*=0.012, 95%*CI*=0.00974, 0.07946); and Alanine Aminotransferase levels (*p*=0.002, 95%*CI*=-332.032, -72.233).

**Conclusion:**

Patients with blood Group-B were more frequent and AB was least commonly affected by the Dengue-virus infections. However, no association was found between a particular blood group and disease severity. Greater proportions of the younger men had Dengue infections.

## INTRODUCTION

Dengue fever is an important arbovirus (DENV) infection found in the tropical and subtropical regions.^1^ It is a mosquito-borne disease, transmitted by *Aedes aegypti*, affecting 390 million people globally.^2^ It is an emerging public health issue in Pakistan. The first epidemic was reported here around 20 years ago and the number of cases continues to increase in recent years.^3^ All four serotypes of DENV have been reported in Pakistan.^4^ Dengue fever is characterized by the presence of fever, headache, myalgias, skin rash, and bleeding diathesis. Altered laboratory parameters include leucopenia, thrombocytopenia, changes in hematocrit, and elevated liver enzymes like ALT and deranged coagulation parameters.^5^-^8^

Dengue virus-induced vascular permeability and plasma leakage is thought to be related to immune-mediated endothelial activation. As blood group antigens are part of innate immunity, it is hypothesized that the antibodies produced against Dengue viral proteins (natural IgM) cross-react with the host cells. Therefore, different ABO blood groups had different susceptibilities to acquiring infections.^9^ There are limited studies regarding the association between Dengue infection and blood groups.^10^ Though some have reported a higher prevalence of various blood groups in Dengue infections; others showed an association of certain blood groups with the severity of Dengue disease.

However, there are discrepancies in the published literature and variability in their results.^11^ The frequency of blood Group-B in DF was shown as 27% by Joshi et al.^12^ A massive Dengue outbreak occurred in recent years in various cities of Pakistan, particularly in Peshawar and Swat districts 13; and cases are still being reported with increased frequency. The purpose of this study was to determine and analyze the frequency of different blood groups in DF patients, and to study the relationship of different blood groups with the severity of the illness. The results will be shared with primary care clinicians, internists, and emergency physicians for appropriate measures in different blood groups with DF and predicting clinical severity in Dengue patients, which may help evaluate patients for their likely need for critical care. It will aid in effectively identifying and managing such cases, which may help in reducing associated mortality and morbidity.

## METHODS

This hospital-based descriptive cross-sectional study was conducted in the Dengue Isolation Ward of the Department of Medicine, Lady Reading Hospital Peshawar, from March 2020 to September 2020. A sample size of 160 was calculated using the World Health Organization software. The formula for “estimating a population proportion with specified absolute precision” was used based on the following assumptions: Confidence Interval (*CI*) of 95%, 7% margin of error, and 27%^12^ anticipated proportion of blood Group-B in patients with DF; five more patients were added to cover for contingency factor.

Patients of both genders aged 14-76 years, fulfilling the criteria for DF, were enrolled in the study after taking informed written consent, by non-probability consecutive sampling technique. Patients who had coexisting malaria, typhoid fever, and leptospirosis; those having co-infection with any other viral hemorrhagic fever (e.g., Chickengunya and ZIKA virus); and those with diagnosed immune thrombocytopenic purpura were excluded from the study to control the confounders. The study was conducted after getting approval from the Institutional Ethical Review Board ERB, Ref: No. 427/LRH/MTI, March 3, 2020.

### Operational definitions

Dengue infection was categorized into three severity levels.

### Dengue Fever (DF)

A patient with a fever of >100^0^F and a positive blood serology test for Dengue NS1 antigen or IgM antibody against Dengue virus was considered as suffering from DF.

### Dengue Hemorrhagic Fever (DHF)

Patients with DF plus bleeding episodes (petechiae, bruises, mucosal bleeds) and evidence of plasma leakage like pleural effusion or ascites.

### Dengue Shock Syndrome (DSS)

Patients with DHF plus evidence of circulatory failure (shock, hypotension with systolic blood pressure<100 mmHg, reduced pulse pressure of ≤ 20 mmHg).

### Blood Groups

The ABO blood grouping system was used for labeling blood groups A, B, AB, and O. Blood Group-A is present when blood agglutinates only on the addition of antiserum against ‘A’ antigen; Group-B is present when blood agglutinates only on addition of antiserum against ‘B’ antigen; Group-AB is present when blood agglutinates on the addition of antisera against ‘A’ and ‘B’ antigens, and Group-O is present when blood does not agglutinate on the addition of antisera against ‘A’ and ‘B’ antigens.

A detailed history was taken from the patients regarding fever, rash, and duration of illness; medical records for any previous Dengue infection in the past. A thorough clinical examination was carried out; vital signs and hydration status were assessed. Blood samples were taken for blood grouping and complete blood count with hematocrit; serum urea, creatinine, Alanine aminotransferase (ALT), and serum bilirubin. The data were recorded and analyzed using SPSS® version 23. Frequencies and percentages were calculated for categorical variables like gender, the severity of illness, and blood groups. Mean and standard deviations were calculated for the numerical variables like age, leucocyte and platelet count, hematocrit, urea, creatinine, ALT, and bilirubin. The frequency of different blood groups was stratified with gender and types/severity of the illness. Post-stratification chi-square (χ^2^) test was applied and a *p*≤0.05 was considered significant. Laboratory parameters of the DF patients were analyzed in both gender groups using Independent Samples *t-*test. Results were presented in the tables.

## RESULTS

Out of 160 patients, 119(74.4%) were males and 41(25.6%) females. The age of the patients ranged from 14 to 76 years, with a mean age of 38.09±15.68 SD, IQR=25 years. Most of the patients (64.4%) belonged to the 21-50 age groups as shown in Table-I. Greater proportion (28%) of the young men (up to 40 years) was affected compared to 9% young women. Fever (99%) and body-aches (96%) were the most common presentation of DF. Shock occurred in 15(9.4%) patients. Thrombocytopenia was found in 137(85.6%) and raised ALT in 119(74.4%) patients. Other hematological and biochemical abnormalities are shown in Table-II. Leucocyte count (x10^3^/mm^3^) ranged from 1 to 37 (mean 7.91±5.28). Mean hemoglobin was 14.22±3.43 gm/dl and hematocrit (x100) ranged from 0.14 to 0.89 (mean 0.41±0.09). Platelets ranged from 4x1000 to 600x1000 with a mean of 51.93±83.84; mean ALT was 188.23±372.71 and mean creatinine was 1.33±1.36. Further analysis of the laboratory parameters using independent sample t-test revealed a significant gender difference of hemoglobin (*t*=2.695, *df* (158), *p*=0.008, 95%*CI*=0.439, 2.844), hematocrit (*t*=2.527, *df* (158), *p*=0.012, 95%*CI*=0.00974, 0.07946); and ALT (*t*=-3.073, *df* (158), *p*=0.002, 95%*CI*=-332.032, -72.233).

**Table-I:** Descriptive statistics of the patients with Dengue fever (n=160)

Variables	Categories	Frequency	Percent
Gender	Male	119	74.4
	Female	41	25.6
Age Groups	14-20	25	15.6
	21-30	38	23.8
	31-40	35	21.9
	41-50	30	18.8
	51-60	17	10.6
	61-80	15	09.4
Dengue Fever Types	DF*	101	63.1
	DHF*	44	27.5
	DSS*	15	09.4
Blood Groups	A	43	26.9
	B	63	39.4
	AB	09	05.6
	O	45	28.1
Rhesus Status	Positive	143	89.4
	Negative	17	10.6
Blood Pressure Category	Normal BP	125	78.1
	Hypotension	10	06.3
	Hypertension	20	12.5
	Un-recordable BP	05	03.1
Symptoms of DF	Fever	158	98.8
	Body-aches	154	96.3
	Bleeding	49	30.6
	Skin Rash	19	11.9
Dengue Serology	NS-1* Positive	160	100
	Ig-M* Positive	06	03.8

* DF: Dengue Fever, *DHF: Dengue Hemorrhagic Fever, *DSS: Dengue Shock Syndrome, *NS-1: Non Structural protein-1 of Dengue virus (DENV), *IgM: Immunoglobulin M Antibody to DENV.

**Table-II:** Hematologic/biochemical Abnormalities in patients with Dengue fever (n=160).

Variables	Categories	Values	Frequency	Percent
Thrombocytopenia (x10^3^/cmm)	Very Severe	less than 10	18	11.3
	Severe	10-29	79	49.4
	Moderate	30-49	19	11.9
	Mild	50-99	21	13.1
	Nil	100 plus	23	14.4
TLC* (x10^3^/cmm)	Low	<4	32	20.0
	Normal	4-11	99	61.9
	High	>11	29	18.1
Hemoglobin (g/dl)	Low	<12	29	18.1
	Normal	12-16	95	59.4
	High	>16	36	22.5
Hematocrit (%)	Low	<35	32	20.0
	Normal	35-45	81	50.6
	High	>45	47	29.4
ALT* (u/L)	Normal	Up to 40	41	25.6
	High	>40	119	74.4
Bilirubin (mg/dl)	Normal	Up to 1.1	117	73.1
	High	>1,1	43	26.9
Urea (mg/dl)	Normal	Up to 40	109	68.1
	Deranged	>40	51	31.9
Creatinine (mg/dl)	Normal	Up to 1.1	111	69.4
	Deranged	>1,1	49	30.6

* TLC: Total Leucocyte Count, *ALT: Alanine Aminotransferase.

The majority (63.1%) of our patients had DF while 27.5% had DHF. There was no significant difference in types of Dengue fever with gender (*p*=0.056). Blood group distribution in DF is shown in Table-III; patients with Group-B were affected in 63(39.4%) of cases while patients with Group-AB were least commonly affected (5.6%). However, further analysis of the blood groups and types of DF showed no significant association between types/severity of DF with a specific blood group (*p*=0.97), as shown in Table-III. The proportion of patients with different blood groups and the type/severity of the DF were almost identical except the fact that none of the patient with Group-AB had DSS (Table III).

**Table-III:** Association of Blood groups with Types/Severity of Dengue fever (n=160).

Blood Groups	Dengue Types			Total

	DF*	DHF*	DSS*	
A	26(60.5%)	12(27.9%)	05(11.6%)	43(26.9%)
B	40(63.5%)	17(27.0%)	06(9.5%)	63(39.4%)
AB	06(66.7%)	03(33.3%)	0(0%)	09(5.6%)
O	29(64.4%)	12(26.7%)	04(8.9%)	45(28.1%)
Total	101(63.1%)	47(29.4%)	15(7.5%)	160(100%)

Pearson Chi-Square (χ^2^)=1.301, df(6), p=0.972.,

* DF: Dengue Fever, *DHF: Dengue Hemorrhagic Fever, *DSS: Dengue Shock Syndrome.

## DISCUSSION

In our study, 74.4% of patients were males with a male-to-female ratio of 3:1. The same trend has been reported by various studies from Pakistan 6, 13-15, and other regional countries.^8^ It can be explained based on increased exposure risk to mosquito bites; as males have more outdoor time compared to females due to occupational and other related activities.^16^ On the other hand, Alves et al.^17^ from Brazil showed no gender differences in dengue patients, which might be due to cultural and social differences. In our patients, there was no significant difference in the types/severity of DF with gender (*p*=0.056). However, children and women had more severe Dengue infections in a study by Yacoub et al.^18^; which can be explained by the difference in sample selection, as fewer females and no pediatric patients were included in our study. The age of our patients ranged from 14 to 76 years with a mean age of 38.09±15.68 years. Most of the patients (64.4%) belonged to the 21-50 age groups. This age group is considered the most active occupationally and socially, thereby more prone to acquiring Dengue infections, as also shown by other local 6,13-15 and regional^8^ studies. Fever (99%) and body aches (96%) were the most common presentation of DF.

Skin rash was found in 19(11.88%) patients; 52.6% of whom had DHF (*p*=0.009). Bleeding occurred in 49(30.6%) patients; 96% of whom had DHF (*p*=0.000) and 15(9.4%) patients presented in shock, exclusively due to DSS (*p*=0.000). Similar clinical features have been reported in other studies.^5^, ^8^ In a local study by Ahmed et al.^19^, skin rash was observed in 28% and bleeding episodes in 13.9%. In the present study, thrombocytopenia was found in 85.6% of patients and severe thrombocytopenia in 11.3%. Ahmed et al.^19^ also reported a mean platelet count of 69290±5082. Low platelet count in Dengue infection is a clinical finding of paramount importance, as thrombocytopenia and coagulopathy may result in bleeding diathesis.^20^ However, the exact mechanism of Dengue infection-related thrombocytopenia is unknown but is considered multifactorial. Although thrombocytopenia is very common in DF patients, however, prophylactic transfusion of platelets to these patients has no benefits.^21^ A comparative study by Lee et al.^22^ in Singapore reported that prophylactic platelets transfusion in DF patients was found to complicate the disease rather than help prevent bleeding.

In the present study, total leucocyte count (TLC) ranged from 1 to 37(7.91+5.28) and hematocrit ranged from 0.14 to 0.89(0.41+0.099). A mean TLC of 4283+1919 and a mean hematocrit of 0.42±4.1 were reported from Peshawar.^19^ Laboratory parameters analysis of our study revealed significant gender difference of hemoglobin (*p*=0.008, 95%*CI*=0.439, 2.844), hematocrit (*p*=0.012, 95%*CI*=0.00974, 0.07946) and ALT levels (*p*=0.002, 95%*CI*=-332.032,-72.233). Other national studies also reported leucopenia, thrombocytopenia, and elevated liver enzymes similar to our study findings.^23^, ^24^ Joshi et al.^12^ from India reported that Dengue patients with blood Group-B had severely deranged laboratory parameters compared to other blood groups. They found severe thrombocytopenia in 74%, leucopenia in 56%, and raised hematocrit in 59% of cases.^12^

The majority (63.1%) of our patients had DF while 27.5% had DHF and 9.4% had DSS. Another study from Rawalpindi/Islamabad 7 reported comparable results to our findings of patients with fever, thrombocytopenia, and raised ALT; as 63% of their patients had DF, 31% DHF, and 6% DSS. Similarly, Riaz et al.^6^ from Karachi also reported thrombocytopenia (60%), leucopenia (45%), elevated ALT (71%), and deranged PT/aPTT. A majority (61%) of their patients had DF, 29% had DHF, and 10% had DSS. A lower incidence of DSS (up to 0.3%) has been shown in healthcare facilities with improved health systems, better resources, and trained and highly professional medical staff .^25^

ABO blood groups were shown to have a potential role in susceptibility to Dengue infections.^9^ Our patients with blood Group-B were affected in 39.4% of cases, while patients with Group-AB were least commonly affected (5.6%) compared to other blood groups. In an Indian study by Ravichandran et al.^10^, 30.7% of patients had blood Group-B. On the other hand, higher frequencies of the ‘O’ blood group of 42.8% and 42% were reported by Khode et al.^11^ and Joshi et al.^12^, respectively.

Contrary to these, blood Group-AB was reported to have an increased frequency of DF as compared to controls (23% vs 8.5%, *p*=0.0004); and blood Group-O was less affected by DF (*p*=0.0048).^23^ In the present study, analysis of blood groups with types/severity of Dengue infection showed no significant association with a specific blood group (*p*=0.97). It conformed to the previously published international studies^10^,^11^, which have shown that all four ABO blood groups had similar susceptibility to Dengue-virus infections; and there was no significant association between disease severity and ABO blood groups.

Our study identified no DSS among patients with blood Group-AB. Although blood Group-B was shown as a predictor of severe Dengue by some studies^26^; however, other studies reported blood Group-AB as a risk factor for severe Dengue infection^27^,^28^, which might be due to higher chances of cross-reactivity of antibodies produced by Dengue infection with both antigens in the AB blood group. Hashan et al.^29^ reported that blood Group-O had the highest risk of developing DF (*p*=0.01) followed by blood Group-B (*p*=0.34). Blood Group-O also had the highest risk of developing DHF (*p*=0.1) followed by blood Group-B (*p*=0.29). Dengue virus infections continue to pose an important threat by challenging the competence of health care providers and capabilities of health authorities in a resource-limited country like Pakistan.

### Study Findings and Practice Implications

The present study revealed that a greater proportion of the young men were affected compared to women. Fever and body-aches were the commonest presenting symptoms of DF. Blood group B was the most affected and AB the least affected blood group by the Dengue virus infections. No significant association was found between types/severity of DF and any particular ABO blood group; however, none of the patients with AB group had DSS. The majority (85%) of the DF patients had thrombocytopenia, 75% had raised Alanine Aminotransferase (ALT) levels. Hemoglobin, hematocrit and ALT levels were significantly different in men and women having DF.

Dengue fever is now in the second decade of endemicity in Pakistan, occurring particularly in summer and autumn. Although the present study showed no significant association with the severity of DF, however, patients with group B having DF needs special attention. All patients with DF with alarming clinical features must be closely monitored for development of complications including DHF, DSS, and multi-organ failure (MOF), and promptly managed. As the treatment of DF is mainly symptomatic and supportive, health education and awareness of the general public and health community regarding preventive measures is the best way to control DF.

### Limitations

It includes a relatively small sample size, descriptive study design, and lack of a control group from the community; and the Dengue serotypes. Further studies are needed to evaluate these parameters.

## CONCLUSION

Patients with blood Group-B were found to be more frequent followed by Group-O. Group-AB was least commonly affected by Dengue virus infections. However, no association was found between disease severity and any particular blood group. Greater proportions of the younger males were exposed to Dengue infections.

### Authors Contribution:

**MARA:** Conceived the idea, designed the study, manuscript writing and final approval.

**ZA, NI & AR:** Did data collection; statistical analysis & manuscript review/editing and final approval.

**MARA and NI:** Accept the responsibility of accuracy and integrity of this work.

All the Authors meet the ICMJE’s requirements for authorship.
